# Construction of an In Silico Structural Profiling Tool Facilitating Mechanistically Grounded Classification of Aquatic Toxicants

**DOI:** 10.1021/acs.est.2c03736

**Published:** 2022-11-29

**Authors:** James W. Firman, David J. Ebbrell, Franklin J. Bauer, Maria Sapounidou, Geoff Hodges, Bruno Campos, Jayne Roberts, Steve Gutsell, Paul C. Thomas, Mark Bonnell, Mark T. D. Cronin

**Affiliations:** † School of Pharmacy and Biomolecular Sciences, Liverpool John Moores University, Byrom Street, Liverpool L3 3AF, U.K.; ‡ KREATiS SAS, 23 rue du Creuzat, ZAC de St-Hubert 38080, L′Isle d′Abeau, France; § Safety and Environmental Assurance Centre, Unilever, Colworth Science Park, Sharnbrook, Bedford MK44 1LQ, Bedfordshire, U.K.; ∥ Science and Risk Assessment Directorate, Environment & Climate Change Canada, 351 St. Joseph Blvd, Gatineau, Quebec K1A 0H3, Canada

**Keywords:** toxicity prediction, aquatic, chemical structure, environmental species, classification, mechanism of action

## Abstract

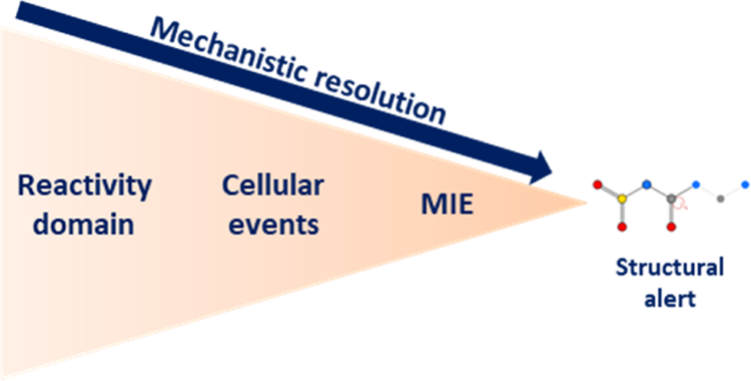

The performance of chemical safety assessment within the domain of environmental toxicology is often impeded by a shortfall of appropriate experimental data describing potential hazards across the many compounds in regular industrial use. In silico schemes for assigning aquatic-relevant modes or mechanisms of toxic action to substances, based solely on consideration of chemical structure, have seen widespread employment—including those of Verhaar, Russom, and later Bauer (MechoA). Recently, development of a further system was reported by Sapounidou, which, in common with MechoA, seeks to ground its classifications in understanding and appreciation of molecular initiating events. Until now, this Sapounidou scheme has not seen implementation as a tool for practical screening use. Accordingly, the primary purpose of this study was to create such a resource—in the form of a computational workflow. This exercise was facilitated through the formulation of 183 structural alerts/rules describing molecular features associated with narcosis, chemical reactivity, and specific mechanisms of action. Output was subsequently compared relative to that of the three aforementioned alternative systems to identify strengths and shortcomings as regards coverage of chemical space.

## Abstract 2

Implementation of the Sapounidou profiler allowing mechanism-based categorization of potential aquatic toxicants, followed by a comparison of chemical space coverage relative to similar schemes.

## 1 Introduction

It is estimated that there are more than 100,000 chemicals in regular industrial use within Europe and North America alone,^1−6^ with three times that number (approximately 350,000) registered globally.^7^ Of these, it is acknowledged that only a small proportion has adequate data, such as a full set of acute hazard and exposure information, appropriate for informing safety decisions.^8^ Due to concerns over potential effects on humans and environmentally prevalent species, there is an increased call for approaches enabling both screening and prioritization of these large numbers of compounds—together with an understanding of mechanisms of toxicity—so that safety assessment might be effectively supported. Computational, or in silico, methods comprise a range of techniques that hold the possibility of providing information on the hazard of chemicals either directly or as part of a weight-of-evidence assessment.^9,10^ Schemes include the Environment and Climate Change Canada Ecological Risk Classification of Organic Substances (ERC, version 2.0), which is a weight-of-evidence logical model relying on data consensus to determine risk classification, risk confidence, and risk severity of organic substances ahead of further regulatory consideration.^6,11^

Within the field of environmental toxicology, computational methods to assess toxicity have commonly taken the form of class-based and mechanistically based quantitative structure–activity relationships (QSARs).^12^ These have been supplemented by read-across and alternative approaches that consider multiple mechanisms or modes of toxic action.^13^ Considering acute environmental toxicity, many such well-established mechanisms of action exist—including nonpolar and polar narcosis, uncoupling of the respiratory electron transport chain, electrophilic reactivity leading to protein adducts, and specific receptor/enzymatic interactions.^14^ Following the pioneering work of Könemann,^15^ further QSARs have been developed based on mechanisms of action.^16^ While many such models can be formulated, their applicability may well be limited—for example, in highly complex (i.e., specific) mechanisms or data-poor chemistries where little information on chronic toxicity exists. In such instances, there is an increasing need to adopt techniques such as read-across as a practical solution. One important application of mechanistically based QSARs and read-across is for regulatory purposes—most notably in filling gaps within the existing data landscape.^17^ If sufficiently transparent, such in silico approaches may offer great utility in terms of meeting regulatory guidelines—assisting in establishing the credibility of alternative approaches in predictive toxicology.^18^ In terms of mechanistically based QSARs and read-across, transparency relates, in part, to the demonstrable linkage of the chemical to that mechanism of action. Recent work has suggested that such mechanistic transparency and justification is a key component of identifying and characterizing the uncertainty of read-across and QSAR approaches.^19−21^

Currently, chemicals may be assigned a mode or mechanism of action on the basis of a number of experimental protocols. The fish acute toxicity syndrome (FATS) method provided a high-quality set of physiological and other responses that can be related to mechanistic understanding.^14^ More recent methodologies have, by contrast, centered upon the use of omics and systems biology approaches.^1,22,23^ However, chemical class or fragment-based systems are still most commonly used both for this purpose and for underpinning the adoption of QSAR and read-across for regulatory applications.^24^ The origin of chemical classification approaches in environmental toxicology may be traced to the 1992 publication of Verhaar et al.^25^—and it is this scheme and its subsequent updates (e.g., Ellison et al.)^26^ which remain perhaps the most widely known and applied. Verhaar’s work was subsequently followed in 1997 by that of Russom et al.^27^ These rule sets, together with MOATox,^28^ have been reviewed and assessed previously by Kienzler et al.—with the conclusion that far beyond their intended purpose in terms of use for relevant species, and are unable to classify a large proportion of chemicals currently in regular use.^24^ More recently, Bauer et al. introduced the MechoA profiler.^29,30^ Drawing from the paradigm of the adverse outcome pathway (AOP)—a concept that has grown to particular prominence over the past decade—this scheme categorized organic chemicals into subclasses anchored not merely in structural similarity but through consideration of molecular initiating events (MIE).^31,32^ Other aims were to provide a common language that can be used by human health specialists and ecotoxicologists for the first time. Subsequently, Sapounidou et al. were to unify and update both Verhaar and Russom protocols with a revised MIE-centered approach, which in turn forms the basis of an attempt to develop an ontology for risk assessment.^33^

Broadly, a distinction may be drawn between the two earlier (Verhaar, Russom) and two later (MechoA, Sapounidou) schemes—and within this work, we shall adopt the phrases “first generation” to refer to the former and “second generation” to refer to the latter. For ease of reference, an overview of the essential characteristics of each is presented in Table S1. The key variation lies within the extent of focus placed upon MIE within the framing of chemical classification, with the increased impetus granted to this within second-generation systems producing assignments rooted more in consideration of the mechanism (rather than merely mode) of toxic action. It is recognized that a number of alternative methodologies for classification of aquatic toxicants exist but these provide neither comprehensive coverage nor a tool that may readily be used in risk assessment. For instance, Barron et al.^28^ described the MOATox system, which is a database of the mode of action classification based on a consensus approach including the Russom scheme and other sources.

While each of the Verhaar, Russom, and MechoA schemes have seen implementation in the form of in silico tools permitting the profiling of chemical libraries, the same has not been true of the Sapounidou rule set. Accordingly, the primary purpose of this investigation was to report the development of an appropriate, freely available resource permitting the practical employment of this scheme in the screening of substances for their potential environmental liability. This takes the form of a workflow within the KNIME analytical software, the core of which lies in a series of structural alerts designed to capture essential chemical features defining participation in the identified MIE. Once operational, this profiler was incorporated alongside those of Verhaar, Russom, and MechoA into a secondary study whereby the performance of each with respect to domain coverage was compared. Particular emphasis was placed upon examining the relative merits of first- and second-generation approaches—the latter of which are anticipated to offer advantages in terms of both the mechanistic resolution afforded and the quantity and breadth of data drawn upon in their development.

The article belongs at the center of a series of works describing the conception, implementation, and progression of the Sapounidou profiler. With early development previously reported,^33^ it is intended that the following step shall see a merger with MechoA to form a unique and comprehensive classification scheme named “MechoA+”.

## 2 Methods

### 2.1 Rendering the Sapounidou Scheme as Structural Alerts and Subsequent Implementation in the Form of KNIME Workflow

A detailed description of the rationale underpinning the construction and content of the Sapounidou scheme is presented in Sapounidou et al.^33^ It should be noted that a selection of minor amendments has since been made to its composition—the majority concerning only terminology. These are referenced explicitly in Table S2.

An overview of the key features within the scheme, as it exists in its present form, is provided in Table 1. In brief, it is structured to incorporate three initial tiers, each offering a progressively enhanced level of mechanistic resolution. Three broad top-level domains are present at Tier 1—“narcosis” (nonspecific effects typically manifesting as “baseline toxicity”), “reactive” (emerging as a product of intrinsic, nontargeted chemical reactivity), and “specific” (targeted interaction at a defined biomolecule, receptor, or pathway). Beneath this, within Tier 2, sit ten mechanistic groups. These are further divided across 25 mechanistic subgroups, together forming Tier 3. Each subgroup is anchored in turn within (potentially several) MIEs, which are themselves defined at the finest level by structural alerts.

**Table 1:** Overview of the Sapounidou Scheme Structure, Incorporating Reference to Those Categories Constituting Tiers 1, 2, and 3^a^

Tier 1 domain	Tier 2 mechanistic group	Tier 3 mechanistic subgroup	no. MIE	no. SA
1. narcosis	1.1. nonpolar narcosis	1.1.1. nonpolar	1	6
	1.2. enhanced narcosis	1.2.1. polar	1	13
		1.2.2. alkyl amine	1	1
		1.2.3. carboxylic acid ester	1	1
2. reactive	2.1. electrophilic	2.1.1. soft	3	32
		2.1.2. hard	7	16
		2.1.3. pre-reactive (electrophilic)	5	26
	2.2. nucleophilic	2.2.1. nucleophilic	1	0
	2.3. free radical generation	2.3.1. radical damage of tissues	1	1
		2.3.2. redox cycling	1	9
		2.3.3. pre-reactive (free radical generation)	1	2
3. specific	3.1. enzyme inhibition	3.1.1. acetylcholinesterase inhibition	1	2
		3.1.2. photosynthesis inhibition	3	8
	3.2. ion channel modulation	3.2.1. modulation of ion channels	8	13
	3.3. cellular function disruption	3.3.1. amino acid biosynthesis disruption	3	6
		3.3.2. cell structure disruption	1	1
		3.3.3. fatty acid biosynthesis disruption	3	8
		3.3.4. nucleic acid biosynthesis disruption	2	2
		3.3.5. steroid biosynthesis disruption	2	5
		3.3.6. carotenoid biosynthesis disruption	3	5
		3.3.7. protein biosynthesis disruption	1	2
		3.3.8. developmental disruption	4	9
	3.4. mitochondrial disruption	3.4.1. electron transport inhib. (specific)	3	6
		3.4.2. electron transport inhib. (nonspecific)	1	2
	3.5. nuclear receptor modulation	3.5.1. modulation of nuclear receptors	2	7

a Further outlined are quantities of MIEs and structural alerts (SAs) corresponding to each.

Implementation to form a practical in silico profiling tool was achieved through construction of a workflow within KNIME analytic software (v.4.3.1; www.knime.com).^34^ This was constituted such that it returns all accompanying information associated with given assignments—including the domain of taxonomical applicability—as presented in Table S3. Structural alerts were compiled from expert knowledge of chemistry surrounding those molecular initiating events established, within the literature, as holding relevance to aquatic toxicology. Their form was tailored such that both excessive exclusivity and generality in terms of potentially matched compounds were minimized. Ultimately, each was coded in the form of SMILES Arbitrary Target Specification (SMARTS) (www.daylight.com). Where possible, rules and alerts were adapted from existing schemes, with adjustments made to ensure coverage of a more appropriate spectrum of chemicals (as supported by existing knowledge). Alerts relating to narcosis were, for example, drawn primarily from Verhaar et al.^25^—supplemented by the addition of rules covering carboxylic acid esters and various forms of ionic and nonionic surfactants.

### 2.2 Analysis of the Sapounidou Scheme as Implemented in the KNIME Workflow

Analysis of Sapounidou scheme domain coverage was performed through screening of an “extended inventory”, consisting of more than 75,000 compounds. The origins of this are described in Table S4. To provide as broad a possible coverage of chemical space and so more effectively identify areas yet uncovered by current rules, substances were drawn from nine publicly available data sets—several of which were specific in terms of use-class and origin. Termed “defined-use inventories”, these included pesticides, pharmaceuticals, botanical natural products, and cosmetic constituents, alongside the European Chemical Agency (ECHA) Registration, Evaluation, Authorisation and restriction of Chemicals (REACH) preregistration list. Chemicals present within each set were subject to preprocessing, within which available SMILES were canonicalized (Open Babel v.2.4.0; http://openbabel.org/wiki/Main_Page),^35^ salt components were stripped, and stereochemical information was deleted. Duplicate entries were removed, alongside inorganics and those lacking defined structures such as mixtures and polymers.

### 2.3 Interscheme Comparison of Domain Coverage

Assessment of domain coverage relating to each of the Verhaar, Russom, MechoA, and Sapounidou schemes was performed through profiling of the “test inventory” of chemical structures. In brief, this list consists of approximately 5500 compounds sourced from the contents of three primary data sets, each of which catalogs substances associated with occurrence in surface water (details are provided in Table S4, as “surface water-relevant inventories”). For further details as regards the properties of chemicals present in this set, please refer to Table S5—within which distributions are provided relating to the spread of molecular weight and logarithm of the octanol–water partition coefficient (log *P*). The latter was calculated within KNIME using the “SLogP” function, accessible through the RDKit “Descriptor Calculation” node (v.4.5; https://www.rdkit.org/). Characterization of structural features was achieved through acquisition of ToxPrint chemotypes generated through ChemoTyper software (version 1.0; Molecular Networks, Erlangen, Germany).^36,37^

The Verhaar rule set was accessed through the OECD QSAR Toolbox^38^ (v.4.4.1; www.qsartoolbox.org), the Russom scheme was accessed through Chemprop (v.7.1.0; http://www.ufz.de/ecochem/chemprop), MechoA was accessed through the MechoA (v.2.2) functionality in the iSafeRat Desktop (v.2.1.0; https://isaferat.kreatis.eu/), and the Sapounidou approach was accessed using the KNIME Workflow described in Section 2.1.

## 3 Results and Discussion

### 3.1 Development of Structural Alerts and Coding into Computational Workflow for Running of the Sapounidou Scheme

**Figure 1:**
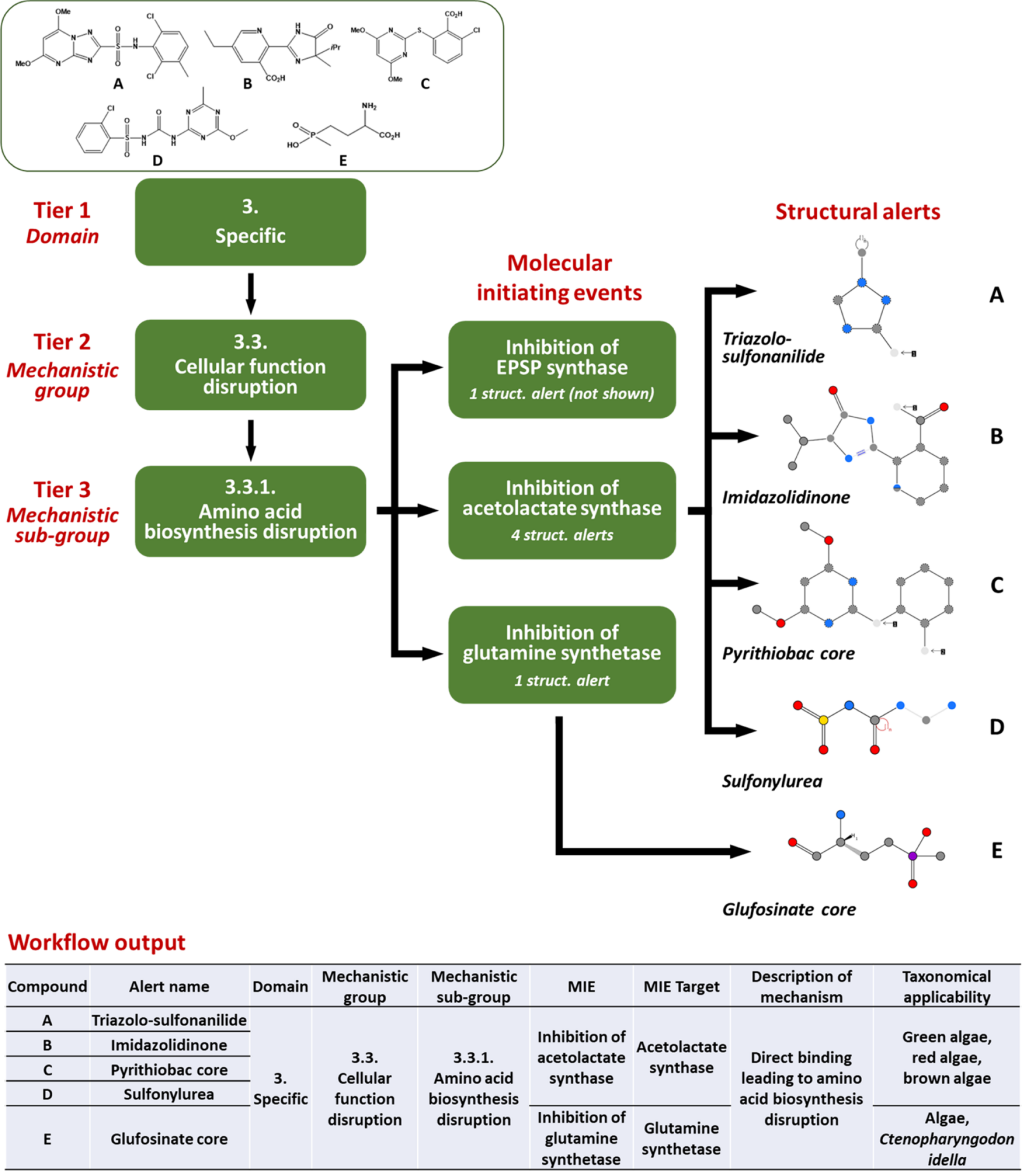
Depiction of Sapounidou profiler functioning as regards screening of five compounds (A–E), each matching a structural alert corresponding to those MIE present under mechanistic subgroup 3.3.1 (amino acid biosynthesis and disruption) within algae. An illustration of output for each, as it would appear following the running of KNIME workflow, is further provided.

Figure 1 provides an illustration of the form of function of the Sapounidou scheme, serving as a general overview of the pathway through which mechanistic assignments are derived from chemistry. The passage of five representative compounds is depicted, each of which matches against a single structural alert associated with the emergence of toxicity in algae as mediated through disruption of amino acid biosynthesis (compound A hitting the triazolo-sulfonanilide alert, B hitting the imidazolidinone, and so on). As evident, three distinct MIEs constitute mechanistic subgroup 3.3.1—each centering upon specific inhibition of a distinct enzyme integral in the derivation of selected amino acids within the appropriate species. While 5-enolpyruvylshikimate-3-phosphate (EPSP) synthase and glutamine synthetase are each represented by a single alert (the former not depicted), allosteric modulators at acetolactate synthase may take one of the four known forms (A–D)—these corresponding to established herbicide classes. KNIME workflow output relating to these substances is additionally provided to serve as an indication of the extent and layout of information provided through the profiler. This tool is freely available for download through links https://github.com/LJMU-Chemoinformatics/Sapounidou-mechanistic-profiler (GitHub) or https://zenodo.org/record/7100972#.YysJ93bMLIU (Zenodo).

Considering the scheme in its entirety, it was necessary to encode a total of 183 structural rules. While in the great majority of instances it was possible to express key chemical features through the use of single SMARTS strings (as in Figure 1), it was necessary that several entries within the narcosis domain be defined by alert sequences. For example, two or three distinct SMARTS were used (stepwise) to define specific groups of surfactants, as illustrated in Table 2 (in this instance, quaternary ammoniums). This differentiation is inevitable due to the nature of the endpoints and is broadly in line with the scheme proposed by Cronin and Richarz^16^ for the capturing of MIEs by in silico methods. For instance, the nonspecific narcosis mechanisms are more readily captured by broad chemical alerts, e.g., representing chemical classes; reactive mechanisms are captured by functional groups relating to organic chemistry reactions. Additionally provided in Table 2 are representative alerts from each of the Tier 1 domains (incorporating one from each mechanistic group under narcosis: nonpolar and enhanced).

**Table 2:**
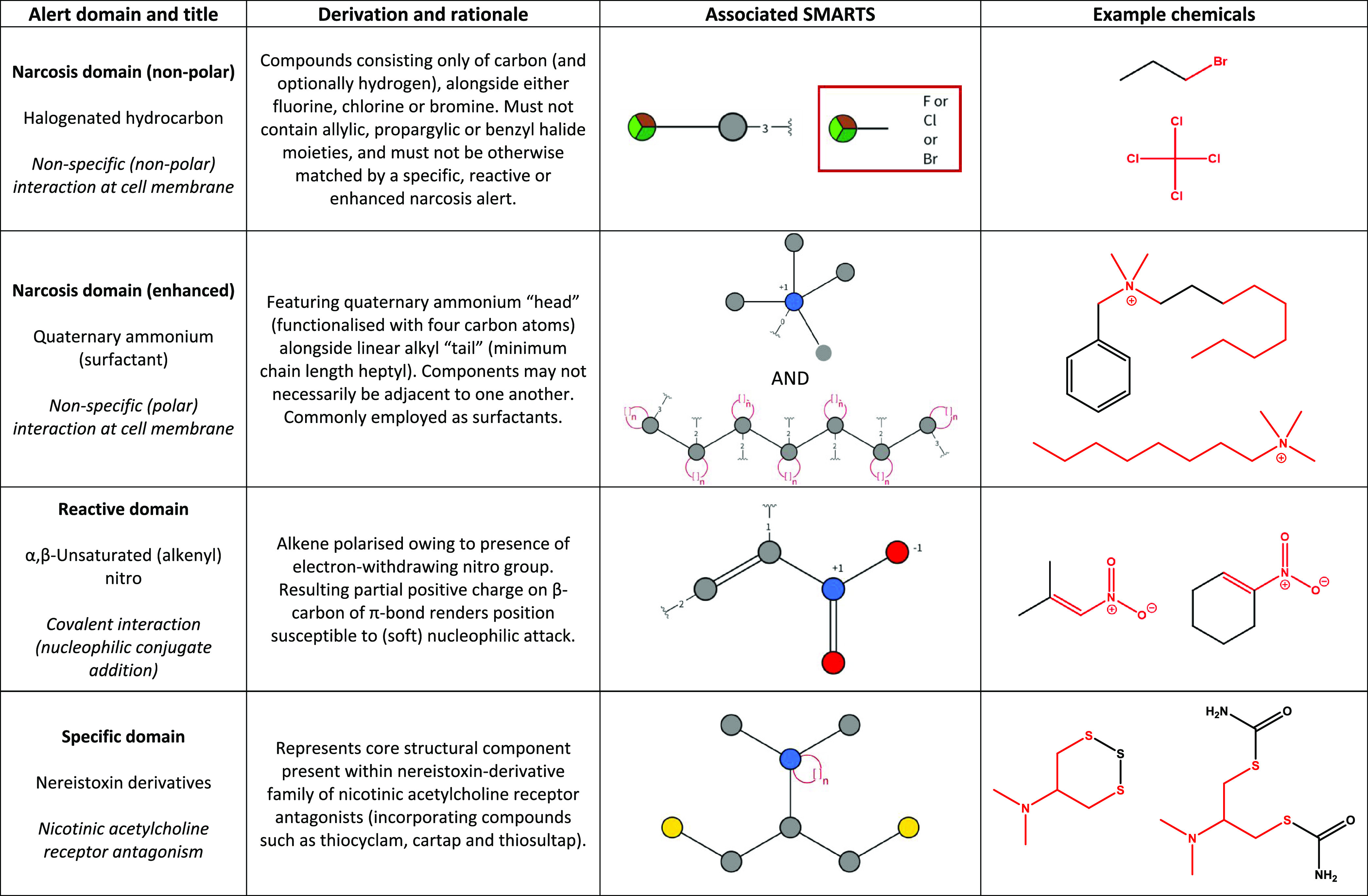
Representative Alerts Drawn from Each Domain Present within the Sapounidou Scheme (Incorporating Two from within “Narcosis”)^a^

a The structural alert is shown in red in the example compounds. Visualization of SMARTS is achieved through the use of the SMARTSview tool (https://smarts.plus/; accessed 1-6-2021) (Schomburg et al., 2010). Key: bromine, brown; carbon, gray; chlorine, light green; fluorine, dark green; nitrogen, blue; oxygen, red; sulfur, yellow.

The KNIME workflow into which these structural alerts were integrated was organized such that rules were applied sequentially, in line with the pathway outlined in Figure S1. Chemicals are initially profiled concurrently using alerts from within the reactive and specific domains—alongside those representing mechanistic group 1.2 (enhanced narcosis). Since compounds are screened in tandem across these domains/groups, it is possible that each may receive multiple assignments drawn from across them all. Substances unmatched during this initial phase are passed through to a secondary stage, in which they are profiled using the rules for nonpolar narcosis. As such, a chemical receiving either a reactive, specific, or enhanced narcosis assignment cannot further match as a nonpolar narcotic.

### 3.2 Coverage of the Sapounidou In Silico Profiler against Chemicals within an Extended Inventory

**Figure 2:**
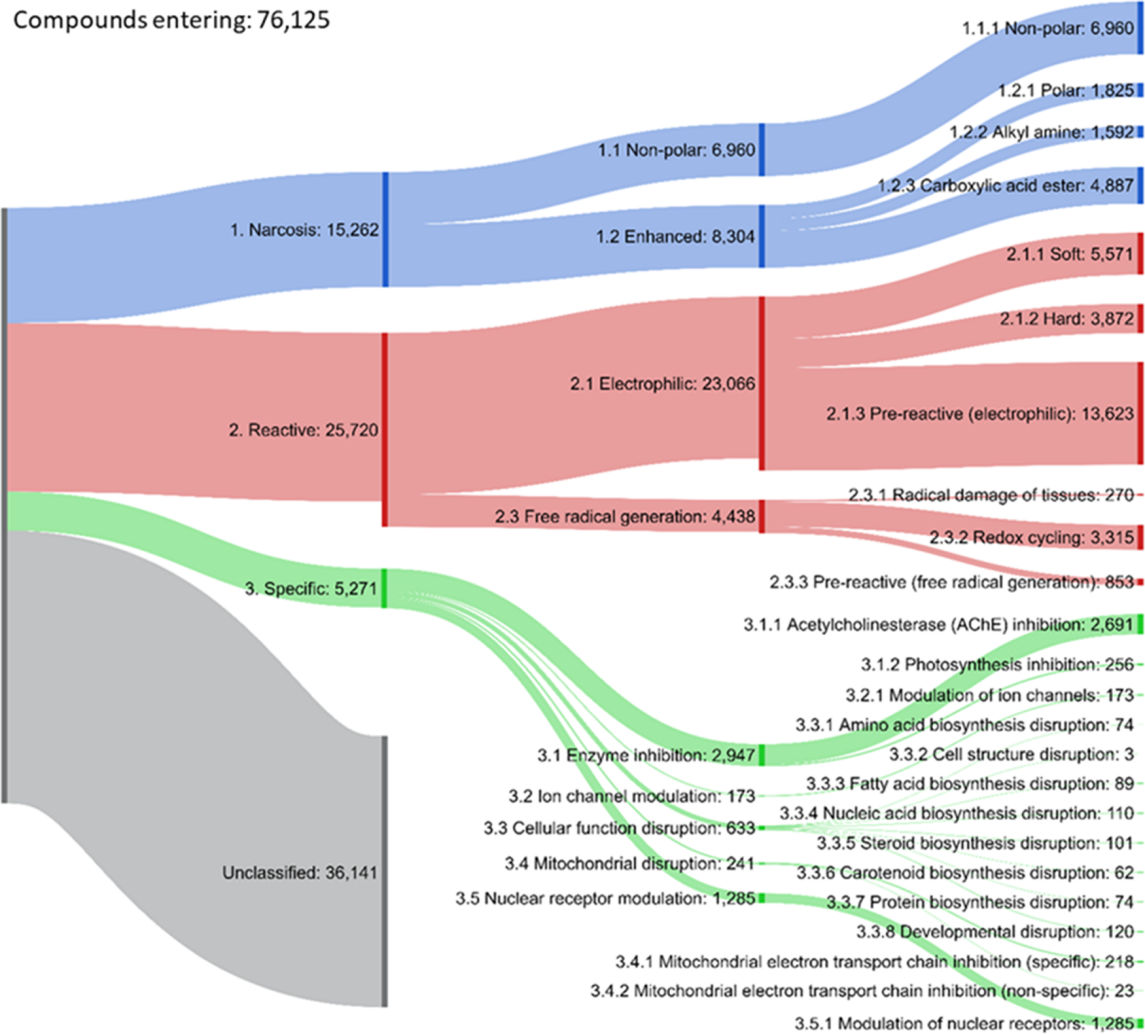
Sankey diagram depicting the quantity of compounds within extended inventory assigned to each classification within the Sapounidou scheme. Note that chemicals may match more than a single group, and as such values relating to lower levels may exceed those in preceding higher tiers. The figure is created using the “SankeyMatic” online tool (https://sankeymatic.com/; accessed 1-12-2021).

To investigate the utility of the Sapounidou scheme as a novel categorization method, it was employed in the screening of an extended compound inventory. Figure 2 shows the outcome of this exercise in terms of the numbers of classifications relating to individual mechanisms. It can be seen that from the 76,125 compounds, 36,141 (47.5%) remained without assignment—these falling outside of the domain of the profiler. Of those assigned, 29,718 were matched against a single alert. A further 7341 were seen to hit two alerts, and the remaining 2925 chemicals were seen to hit three of more (up to a maximum of 12). The current scheme is intended to identify any potential alert that is associated with a MIE rather than to provide a definitive answer to the mechanism of action involved. Assignment to a mechanism of action may be required for certain purposes, e.g., classification and labeling, but is not the remit of the development of the in silico profiler itself. For this purpose, it may be advisable to use the Sapounidou scheme in combination with another tool that unequivocally assigns a mechanism of action class—typically the second-generation MechoA profiler. This would allow drawing a consensus conclusion regarding the most relevant or probable mechanism. Grouping of out-of-domain substances would be achieved by either chemical similarity or on functional groups—although lacking of course the mechanistic basis. Among those 39,984 chemicals matching at least a single alert, 53 differing taxonomical categories were covered. These ranged from universal (across “all taxa and species”), through domain (Eukaryota), phylum (Arthropoda), and ultimately to individual species such as *Daphnia magna* and *Danio rerio*.

**Figure 3:**
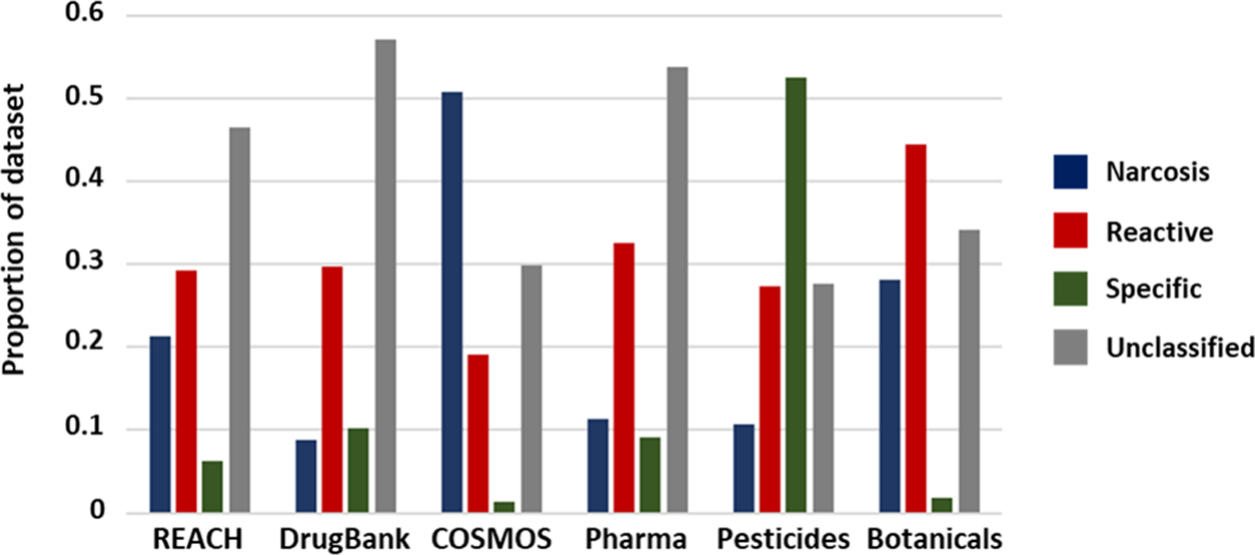
Analysis of the extent of coverage offered by THE Sapounidou scheme across representative compound inventories, presented at the Tier 1 (domain) level.

To provide greater resolution with regard to the functioning of the scheme against chemicals holding similar properties, a further six defined-use inventories (as outlined in Table S4) were individually screened. The proportion of the data sets identified as belonging to each Tier 1 domain are shown in Figure 3. Profiling of the inventory related to cosmetics (COSMOS) revealed that a majority of chemicals (50.8%) fall within the domain of narcosis.^39^ This is largely expected since the inclusion of noninert chemicals within such products would generally be considered highly undesirable. Accordingly, those constituting the set tend to be small in size, possessing only simple functional groups. By contrast, a significant number of compounds within the pesticide dataset (52.5%) are assigned to the specific domain. This is again anticipated, as a number of specific mechanisms of action relating to pesticides were captured and integrated into the workflow. Sharing a related chemical space, it is not surprising to observe that DrugBank and Pharma data sets exhibit similar distributions of coverage—with roughly 10% of substances in each matching narcosis or specific alerts, a further 30% assigned reactive, and 55% failing classification. This highlights a necessity to broaden the range of the mechanisms of action presently detected by the workflow within the specific domain, currently dominated as it is by pesticide modes. With the capacity of pharmaceuticals to exert off-target, adverse effects against several species falling within the remit of this scheme, the integration of such mechanisms would appear to be a rational progression.

### 3.3 Interscheme Comparison of Domain Coverage

To enable direct comparison between the four schemes in terms both of raw coverage and concordance in domain assignment, the test inventory (as described in Section 2.3) was profiled through each. For the purposes of this investigation, extent of “raw coverage” relates to the proportion of chemicals not receiving an “unclassified” assignment (e.g., to Class 5 within Verhaar). It should be noted that the Russom protocol assigns by default the status of “narcotic” to all compounds, not otherwise matching an alert across its alternative domains. As such, no entry is formally “unclassified”, and its outputs cannot be used within this strand of analysis. However, its ChemProp implementation does state whether a compound sits inside or outside the applicability domain. It is apparent that MechoA provides the highest extent of coverage (assigning 5074 of total OF 5517 compounds). The Sapounidou scheme (assigning 3165 substances), however, offers improvement relative to the Verhaar (2369 compounds) profiler. Only 1096 (19.8%) of molecules within the inventory are adjudged to fall within the defined Russom applicability domain. By contrast, 3667 and 754 substances are labeled, respectively, as lying definitively and borderline beyond. Among all other schemes, those substances receiving classification are held by default to fall entirely within their respective domains.

**Figure 4:**
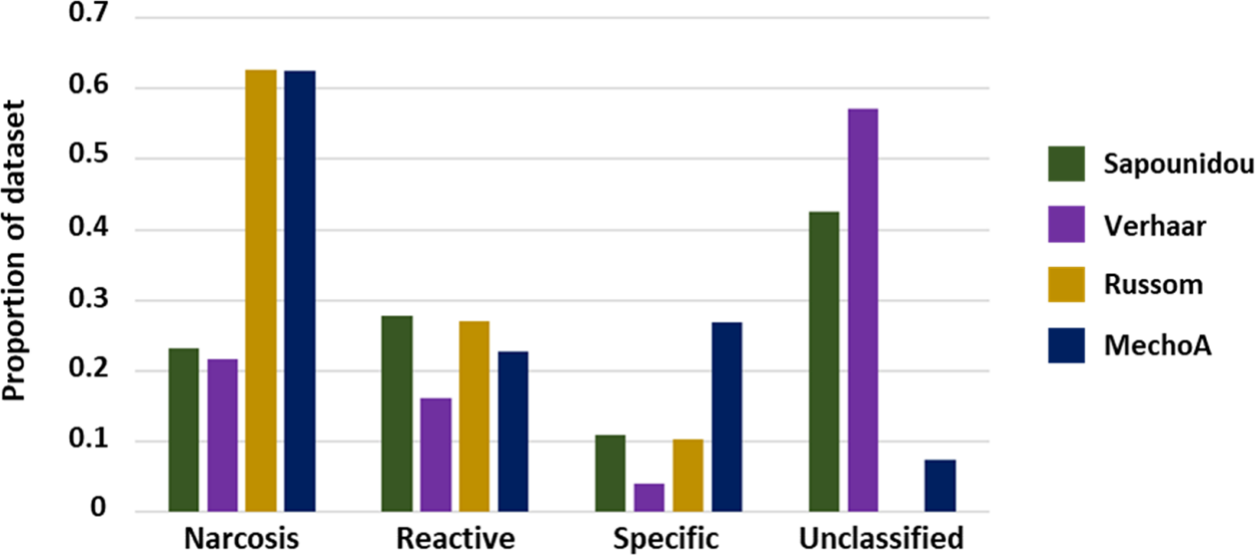
Comparison of domain-level mechanistic classifications (test inventory substances) across each scheme.

To compare the mechanistic assignments of the schemes, subgroups present across each were collated and mapped as belonging either to the narcotic, reactive, or specific modes—or alternatively as being unclassifiable or out of domain. Details of this mapping are presented in Table S6: as such, a compound assigned Verhaar class 3 would align to a “reactive” domain, Russom class 6 would align to a “specific” domain, and MechoA 1.3 would align to a “narcotic” domain (and so on). It is important to note that, as is the case with the Sapounidou scheme, chemicals may receive multiple mechanistic assignments through MechoA (e.g., different MechoAs for different species). By contrast, Verhaar and Russom profilers produce single verdicts. Each rule set was employed to profile the test inventory, with the proportion of classifications derived shown in Figure 4 (in instances where multiple assignments are granted to a single chemical, each is considered distinctly). MechoA and Russom schemes are seen to assign most compounds to the narcotic domain. However, it should be remembered that the implementation of the Russom profiler judges compounds narcotic by default if no other alerts are hit, and as such, this may mean that the domain is overemphasized. As noted in Figure 4, the Verhaar scheme displays the lowest coverage, especially for specifically acting compounds. Further analysis dedicated to assessing the extent of overlap (or alternatively disagreement) with respect to domain-level classification between Sapounidou and alternative schemes is presented in Table S7.

### 3.4 Examination of Unclassified Chemical Space

Despite improvements noted in recent schemes, a proportion of the chemicals within each was nevertheless assigned as either “unclassified” or “out of domain”. MechoA performed best in terms of the absolute number of chemicals classified. To better understand the chemical space of substances unclassified by the Sapounidou profiler, it was necessary to draw upon corresponding information sourced from those alternative rule sets. As a fellow second-generation system, the MechoA scheme is most appropriate for comparison. MechoA assignments were particularly useful for purposes of investigating chemical space—given the level of detail supplied and the large proportion of chemicals for which it could definitively attribute mechanism of action. Note that of the 2352 chemicals “unclassified” through the Sapounidou profiler, 373 were similarly unassigned through MechoA (resulting in 1979 chemicals receiving a classification from MechoA and not from the Sapounidou scheme).

The primary domain for which the Sapounidou scheme shows a reduced extent of classification relative to MechoA is that of narcosis. Rules defining narcosis within the Sapounidou profiler draw extensively from those presented by Verhaar et al.^25^ Limitations, however, are present with respect to the extent of chemical space actively covered. For example, conditions governing the assignment of phenols to this domain restrict the range of permitted compounds only to those “weakly acidic” monohydroxybenzenes further substituted with chlorine, alkyl, or (lone) nitro groups. As such, a vast array of potentially eligible substances evade labeling. Simple, unactivated nitrile compounds (alkyl or aryl) are similarly overlooked—as are sulfur-containing molecules. Our reworking of the Verhaar rule concerning the nonpolar narcosis of chemicals containing only carbon, hydrogen, and a halogen furthermore led to the inappropriate exclusion of a number of aryl halides. It is our intention that these deficiencies shall be rectified in future iterations of the scheme—and to this end, integration alongside MechoA (thus forming MechoA+) is proposed. Table 3 presents the examples from the groups specifically referenced above—alongside a listing of the quantities of chemicals among the unclassified 2,352 that would be expected to meet the relevant inclusion criteria (dominant MechoA assignment is additionally provided).

**Table 3:**
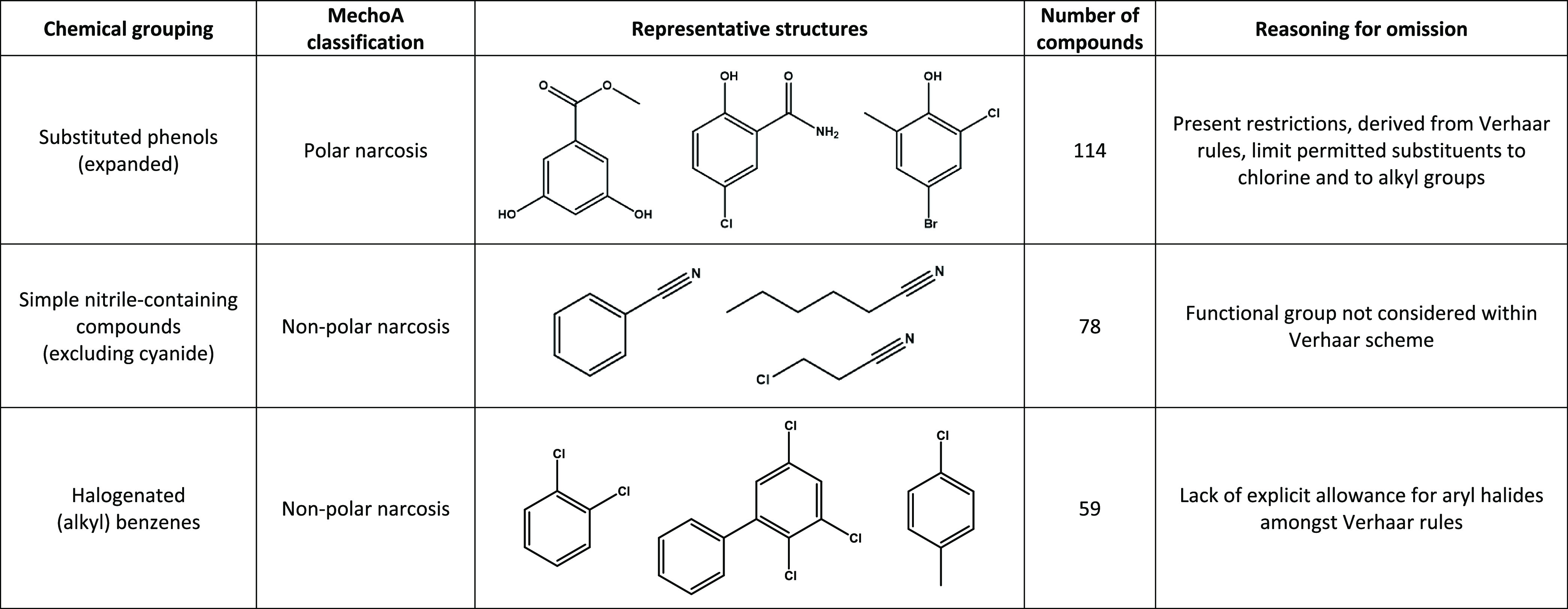
Illustrative Classes of Chemicals Present within the Test Inventory yet Lying beyond the Domain of the Sapounidou Profiler in Its Present Form^a^

a “Number of compounds” relates to the quantity of those 2352 unclassified substances matching the appropriate description.

### 3.5 Current Status of the Sapounidou Scheme among Landscape of Alternative Profilers

Reported within this study is the construction and subsequent performance assessment of a mechanistically grounded in silico profiling tool (freely available as a KNIME workflow) enabling environmental toxicant classification, as derived from the rule set recently published by Sapounidou et al.^33^ Analysis is framed in relation to its strengths and present shortcomings when judged against similar existing schemes, both of the first generation (Verhaar and Russom profilers) and the second generation (MechoA).

It is apparent that, in its present form, the proportion of compounds unclassified remains excessive (for reasons explored in Section 3.4). Nevertheless, it is intended that this situation may be readily rectified moving forward—and as such, the integration alongside MechoA (yielding MechoA+) is an ongoing process. Already, Sapounidou offers the most extensive coverage with respect to the reactive domain and further incorporates a wide array of alerts relevant to specific actions. By defining the structural features in greater detail, and by reducing the number of unclassifiable results, an increased coverage of chemical space and broader domain of applicability shall emerge. This allows for more extensive application by users and is particularly useful when profiling large chemical inventories in which chemical space is inherently wide.^40^

As a second-generation classification system, in common with MechoA, its assignments are anchored at the level of MIE—and therefore by extension to AOP, in instances where such links are established. These schemes in particular support the growing desire to reduce animal testing by providing in silico tiers for Integrated Approaches for Testing and Assessment (IATA) related to mechanistic toxicology.^41^ Their output may, for example, help to strengthen and populate AOPs currently being gathered by the OECD via the AOPWiki initiative.^42^ Through enabling closer linkage of chemistry (e.g., via SMARTS) with biology, sound reasoning for structural alerts associated with potential baseline and nonbaseline toxicity across species may be established. This can be used for better understanding interspecies variation—a variable often used when determining the magnitude of assessment or uncertainty factors used in ecological risk assessment.

This increased transparency with regard to the basis of chemical interactions at the site of toxicity may be of further benefit in prioritization and risk assessment of compounds for which there is a greater desire to explain and predict toxicological outcomes, particularly when traditional in vivo data may be lacking. Inclusion of well-substantiated mechanistic information may help to provide weight of evidence for risk assessment where it is desirable to treat specifically acting chemicals (as opposed to nonspecific narcotics) with more caution when deriving predicted no-effect concentrations^43^—or similarly when prioritizing hazard or risk of substances for further regulatory actions such as that performed by version 2.0 of the Ecological Risk Classification of Organic Substances approach.^6,11^ During problem formulation or prioritization stages, a sound understanding of the modes and mechanisms of action helps to scientifically rationalize the formation of structurally and mechanistically similar chemical categories (groups). These categories may then be adopted to conduct read-across or perhaps category-based risk assessment, including cumulative approaches if applicable. Second-generation schemes may also find application in eco-conception, i.e., in the process of the development of new chemicals, as the first screening of potential hazards before substances are synthesized on a large scale and go through regulatory dossiers.

## Supporting Information Available

Outline of the structure underlying the KNIME implementation of the Sapounidou scheme (Figure S1); overview and comparison of the structure of referenced schemes designed for classification of chemicals according to the mode/mechanism of action relevant in environmental toxicology (Table S1); current iteration of the scheme as compared with that presented in Sapounidou et al. (Table S2); current iteration of Sapounidou mechanistic classification scheme (Table S3); identity, origin, and composition of representative chemical screening inventories (Table S4); general physicochemical and structural characteristics of compounds present within test inventory (Table S5); alignment of classifications from Sapounidou, Verhaar, Russom, and MechoA schemes, as framed within the three Tier 1 domains (Table S6); and analysis of the extent of overlap/variation noted between schemes with respect to the domain-level classification of test inventory (Table S7) (PDF)
